# Family Climate as a Mediator of the Relationship between Stress and Life Satisfaction: A Study with Young University Students

**DOI:** 10.3390/bs14070559

**Published:** 2024-07-02

**Authors:** Paula Morales Almeida, Cristina Nunes

**Affiliations:** 1 Department of Psychology, Sociology and Social Work, Universidad de Las Palmas de Gran Canaria, 35001 Las Palmas de Gran Canaria, Spain; paula.morales@ulpgc.es; 2 Research Center of Psychology (CIP), Universidade do Algarve, Campus de Gambelas, 8005-139 Faro, Portugal

**Keywords:** college student, family climate, perceived stress, life satisfaction, mediation

## Abstract

Background: The family environment has a significant influence on the personality development of young people and their assessment of life satisfaction. Perceived stress is related to life satisfaction, but this relationship is also influenced by the family context. The present study analysed the impact of perceived stress on life satisfaction and the mediating role of family climate in university students. Methods: A sample of 920 university students was recruited for this study. The instruments used included the Perceived Stress Scale, the Satisfaction with Life Scale, and the Family Social Climate Scale, and socioeducational information was collected. Results: The results indicate that perceived stress had a significant and positive effect on both conflictive and violent family climates, while life satisfaction was negatively impacted by these negative family climates. Additionally, supportive and cohesive family climates, along with difficulty expressing feelings and opinions, were found to have significant positive effects on life satisfaction. Conclusions: These findings highlight the importance of the family system in shaping individual conditions and how it can regulate the relationship between stress and life satisfaction.

## 1. Introduction

One in five university students face mental health challenges, representing a notable fivefold increase over the past decade [1]. Furthermore, these students demonstrate diminished overall well-being, as indicated by lower levels of life satisfaction and fulfilment compared to the general population [2,3].

In the context of psychosocial development, stress plays a pivotal role in individuals’ lives. Stress is defined as an organism’s response to physical or psychological demands [4]. Other authors [5] have posited that the severity of stressful circumstances is intrinsically linked to personal development. However, Cohen et al. [6] emphasized that the subjective perception of such events adds complexity to this relationship, underscoring the importance of exploring how individuals experience and manage stress in their daily lives [7]. This perceived stress has been associated with various aspects, including quality of life, resilience, intimate partner violence, and emotional exhaustion [8,9,10,11].

At university, students are immersed in a crucial developmental process, facing significant opportunities and challenges [12,13]. The transition to independent living during this stage can bring about substantial changes, affecting economic, occupational, and residential aspects, consequently generating varying levels of stress [14].

Concurrently, life satisfaction, defined as individuals’ cognitive judgment of their own lives, encompasses ideals, social comparisons, aspirations, and needs [15]. This essential component of subjective well-being becomes a crucial indicator of psychological and emotional adjustment during youth [16]. Factors such as resilience, family support, and intrafamily relationships stand out as significant determinants of life satisfaction in the university context [17,18,19]. Studies have found that self-efficacy and social support are related to life satisfaction in adolescents [20]. Additionally, comparing levels of life satisfaction in young individuals can serve as a predictor of the development of psychological factors, contributing to the enhancement of perceived happiness in young adults [13].

Therefore, perceived stress is correlated with negative emotions such as anxiety and depression, and, most importantly, with lower levels of life satisfaction [21]. In this regard, empirical studies have found a significant negative correlation between stress and life satisfaction among university students in India [22], as have Hasanin et al. [23] with university students in Egypt.

The family is a dynamic social system that responds to both external and internal changes, extending beyond individual personal and psychological qualities such as stress and life satisfaction. University students still financially depend on their parents while simultaneously beginning to share adult responsibilities within the family [24]. It is crucial to consider the relationships among family members and how they influence each other [25], hence the importance of considering the family climate [26].

In this scenario, family climate, understood as the emotional and psychological atmosphere experienced within the household, emerges as a crucial factor in the well-being equation. The various dimensions of family climate, from conflict and violence to support and emotional expression, have been linked to crucial psychosocial outcomes such as life satisfaction [27,28]. Specifically, a conflictive family climate is characterized by tense and hostile relationships among family members, which can hinder the psychosocial development of the individual [27]; a climate of support and togetherness refers to a positive family environment that fosters understanding among family members, encouragement, and reasonable demands, essential for personal development [28]; difficulty expressing feelings and opinions can influence the family climate by generating tensions and conflicts [28]; and a violent climate is characterized by the presence of physical or verbal aggression among family members, leading to a hostile environment and adversely affecting the psychosocial development of individuals [27,29].

In university students, the family climate not only influences relationships and life experiences but impacts academic performance [30]. Enjoying good moments with the family and receiving fair treatment from parents and caregivers have significant and positive effects on life satisfaction in adolescents [31]. However, it has also been acknowledged that a negative family climate can increase stress and decrease life satisfaction [22,32]. Bulo and Sánchez [33] indicated that stress affects university students, especially when they fail in their academic activities, with parental approval being a determining factor for their success. Relationships with family—when not positive—can represent sources of stress for young university students [34].

According to Bronfenbrenner’s ecological systems perspective, human development is best understood by considering the multiple layers of influence surrounding the individual, ranging from the immediate microsystem (such as family and school) to the broader systems like the cultural macrosystem [35]. This theory emphasizes the importance of interactions between different contexts and how these influence individual development and well-being.

Bronfenbrenner [35] posits that the development of university students is mediated by the interaction of personal and contextual elements. In this regard, family characteristics, such as emotional support and cohesion, are fundamental, but so too are interactions with other systems like the educational system and the community [36]. The family context does not operate in isolation; its influence is modulated by factors such as peer relationships, the quality of education received, and prevailing cultural norms [37].

Therefore, to fully understand the impact of stress and life satisfaction on university students, it is essential to consider not only personal and family characteristics but also how these elements interact with broader contexts [35].

### The Present Study

The family plays a key role as a source of social support for university students when facing stress [38]. Barraza [18] found that the family support network and intrafamily relationships are predictors of life satisfaction. Therefore, the family system influences the regulation of personal characteristics. Both stress and life satisfaction are personal aspects related to the family climate. The objective of this study, however, was not to analyse how different contexts influence personal conditions but rather to examine the extent to which they are related to personal conditions. By doing so, we wanted to determine whether family characteristics can improve or impair personal conditions.

However, a deeper understanding of the relationship between family characteristics and personal traits is necessary, as it would enable the identification of family elements that can be targeted by social policies to enhance individual development. Thus, the present study examined the role of the family climate as a component of the dynamics of the family system in the relationship between perceived stress and life satisfaction, considered as personal characteristics. Six hypotheses were tested:

**H1.**  *Perceived stress is directly linked to conflictive and violent family climates.*

**H2.**  *Perceived stress is indirectly associated with positive family climates characterized by social cohesion and emotional expression.*

**H3.**  *A violent and conflictive family climate is indirectly associated with life satisfaction.*

**H4.**  *A positive family climate characterized by social cohesion and emotional expression is directly associated with life satisfaction.*

**H5.**  *Perceived stress is related to life satisfaction.*

**H6.**  *The mediation of the family climate helps to better explain the relationship between perceived stress and life satisfaction.*

**Figure 1:**
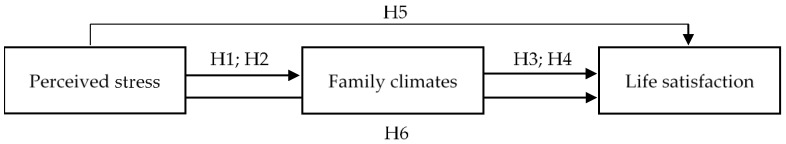
Theoretical model of mediation and hypotheses.

In this study, since the characteristics of the family system can coexist within the system, each family climate was considered as a mediating variable [39]. Thus, a parallel mediation model was tested to analyse the effect of the types of family climate on the relationship between perceived stress and life satisfaction following the theoretical model in Figure 1.

## 2. Materials and Methods

### 2.1. Participants and Procedure

A representative sample of undergraduate students at the University of Las Palmas de Gran Canaria (Spain) was selected using the quota method. A proportional allocation was carried out with a two-stage procedure, with a first stage of distribution of surveys according to conglomerate (grades) and a second stage according to quotas (sex), selecting the last units (students) by random routes in the different campuses of the university. Thus, for a theoretical sample design, a confidence level of 95.5% (two sigmas) was estimated, and a probability of *p* = q = 50% of heterogeneity, obtaining a theoretical sample size of 900 students with a margin of error of 3.23. The sample obtained was 941 students, with an estimated error of 3.15.

It was verified that the variables did not have scores out of range (accuracy) and that there were no missing data. Multivariate outliers were removed using indices from Mahalanobis distance, Cook’s distance, and leverage [40]. Values were identified as outliers if the Mahalanobis distances were above the critical value for five predictor variables, with a probability of 0.001 × 2 = 20.52 (n = 10). The Cook’s distance criterion would imply outlier values greater than Di = 0.0043 (n = 52). And leverage for cases with values above 2 times the number of predictors plus 2 according to the sample size, hii = 0.0137 (n = 52). Finally, two of the three criteria for the identification of outliers were assumed (n = 20). The final sample of the study consisted of 920 university students, with a mean age of 21.32 years (SD = 3.54) and of which 54.3% were women (see Table 1).

The minimum sample size was calculated [41] using G*Power version 3.1.9.7 [42], an a priori power analysis, which calculated that a sample of 159 participants was required for a small effect size (f^2^ = 0.05), for two tails, with an alpha of 0.05 and four predictors, to achieve a power of 0.80. However, estimating the sample size for mediation models has the difficulty of estimating the indirect effect, even using specific tools such as MedPower version 2.0 [43] when using only one mediator variable. Thus, the current study targeted a considerably greater sample size than this estimate.

Our questionnaire was developed and prepared in an online format. Four surveyors were trained for fieldwork. An online access link to the survey was distributed to conduct face-to-face interviews with university students. Participants were informed about the study’s objective, requested voluntary participation, assured anonymity, and required to provide informed consent. The responses were consolidated into a common database. The data collection took place on the campuses where the undergraduate programs were located between 24 March and 2 May 2023, with an average execution time of 15 min.

### 2.2. Measures

#### 2.2.1. Perceived Stress

Perceived stress was assessed using the Perceived Stress Scale [6] adapted to Spanish by Remor [44], with an adequate Cronbach’s alpha (α = 0.81). The scale consists of 14 items (e.g., “In the past month, how often have you been upset because of something that happened unexpectedly?”; “In the past month, how often have you felt unable to control the important things in your life?”), with a 5-point Likert scale ranging from never (0) to very often (4). Higher scores indicate higher levels of perceived stress.

#### 2.2.2. Life Satisfaction

Life satisfaction was analysed using the Satisfaction with Life Scale by Diener et al. [45], adapted by Atienza et al. [46], with high reliability (α = 0.84). The scale consists of five items (e.g., “In most aspects, my life is as I want it to be”; “The circumstances of my life are very good”), with five response options ranging from totally disagree (1) to totally agree (5). Higher scores reflect greater life satisfaction.

#### 2.2.3. Family Climate

The adapted Family Social Climate Scale by Moos et al. [47], as revised by Marchena et al. [48], was used. It includes 24 items (e.g., “In my family, not a day goes by without some argument”; “There is a strong sense of unity in my family”; “In my family, we usually resolve problems through discussion”; “In my family, sometimes we get so angry that we hit or break something”), with five response options ranging from totally disagree (1) to totally agree (5). The scale exhibits good fit indices: RMSEA = 0.069; CFI = 0.96; TLI = 0.95; WRMR = 1.6, with a reliability of α = 0.96. The explored factors are Conflictive Climate in the Family (seven items, α = 0.89), Supportive and Cohesive Climate (10 items, α = 0.90), Difficulty Expressing Feelings and Opinions (five items, α = 0.81), and Violent Climate in the Family (two items, α = 0.69).

#### 2.2.4. Socioeducational Information

The study included questions about age (continuous), gender (dichotomous), as well as the field of study (four categories) and academic year (ordinal, four categories) of the participants.

### 2.3. Data Analysis

The data were analysed using IBM’s Statistical Package for the Social Sciences (SPSS, Version 27). Descriptive statistics were calculated for demographic data and study variables. The associations between variables were calculated using Pearson’s bivariate correlation, following the indication of the strength of association according to Cohen [49]. The significance level was set at 0.05 in all analyses.

The values of kurtosis and asymmetry for the study variables were examined, specifying a range ±1.5 (asymmetry, between −0.412 of life satisfaction and 0.631 of Violent Climate, and kurtosis, between −0.052 of perceived stress and 0.664 of the climates for expression difficulties), and compliance with the assumption of normality was observed [40].

The extreme values were determined by calculating the Mahalanobis distance, Cook’s distance, and the leverage statistic. One participant with extreme values based on two of these three parameters was excluded from the data set.

For the effects of parallel mediation, the bias-corrected bootstrap method was used by estimating the confidence interval (CI), with statistically high efficacy and precision [50]. This study used the PROCESS macro (Model 4) in SPSS with perceived stress as the independent variable, life satisfaction as the outcome variable, and the four dimensions of family climate as mediators (Conflictive Climate in the Family, Supportive and Cohesive Climate, Difficulty Expressing Feelings and Opinions, and Violent Climate in the Family). The significance of the indirect effects was assessed using bias-corrected CIs estimated from 10,000 simple bootstrap samples and was considered statistically significant if the 95% bias-corrected CI did not contain 0 [51].

## 3. Results

### 3.1. Descriptive and Correlational Analyses

Table 2 presents a description of the study variables. Overall, the students reported a moderately lower level of perceived stress compared to the mean of the scale (M = 26.44, SD = 7.44) and a level of life satisfaction above the mean of the scale (M = 18.45, SD = 3.41). Regarding the different family climates, higher levels were reported for the climate with Difficulty Expressing Feelings (M = 3.07, SD = 0.52) and Supportive Climate (M = 2.92, SD = 0.37), while Violent Climate had the lowest reported level (M = 1.9, SD = 0.72).

Life satisfaction was strongly and negatively associated with perceived stress (r = −0.49, *p* < 0.01) but positively and moderately correlated with Difficulty Expressing Feelings (r = 0.21, *p* < 0.01). Additionally, Violent Climate in the Family was positively and moderately correlated with Conflictive Climate (r = 0.32, *p* < 0.01).

### 3.2. Parallel Mediation Model

**Figure 2:**
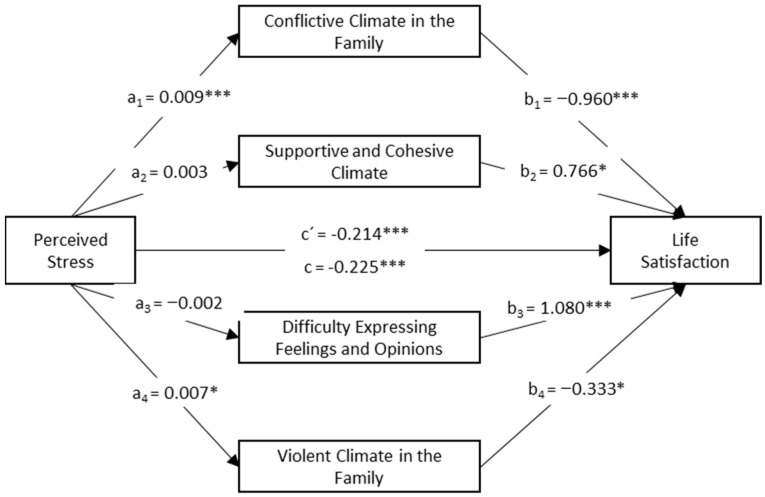
Parallel mediation model of family climates in the relationship between perceived stress and life satisfaction (N = 920). Note: * *p* < 0.05, *** *p* < 0.001.

The results of the parallel mediation model, analysing the effect of different family climates on the relationship between perceived stress and life satisfaction, are shown in Figure 2. Perceived stress had a significant and positive effect on Conflictive Climate (β = 0.009, t = 4.911, *p* < 0.001, IC of the 95%= [0.006, 0.013]) and Violent Climate (β = 0.007, t = 2.132, *p* = 0.033, IC of the 95%= [0.001, 0.013]). For each unit increase in perceived stress, there was an increase in the conflictive and violent family climates.

Life satisfaction was negatively and significantly predicted by Conflictive Climate in the Family (β = −0.960, t= −3.730, *p* < 0.001, IC of the 95% = [−1.465, −0.455]) and Violent Climate (β = −0.333, t= −2.387, *p* = 0.017, IC of the 95% = [−0.607, −0.059]), and positively and significantly predicted by Supportive and Cohesive Climate (β = 0.766, t = 2.360, *p* = 0.018, IC of the 95% = [0.129, 1.404]) and Difficulty Expressing Feelings and Opinions (β = 1.080, t = 5.003, *p* < 0.001, IC of the 95% = [0.656, 1.504]). For each unit increase in the conflictive or violent family climates, there was a decrease in life satisfaction. Similarly, for each unit increase in the Supportive and Cohesive Family Climate and the Difficulty Expressing Feelings and Opinions factors, there was an increase in life satisfaction.

Table 3 displays the different effects of the parallel mediation model. The direct effect of perceived stress on life satisfaction was significant (β = −0.214, t = −16.598, *p* < 0.001), and the total effect indicates a significant model explaining 24.1% of the total variance in life satisfaction (β = −0.225, t = −17.074, *p* < 0.001).

The total indirect effect was significant (b = −0.011, SE = 0.004, 95% CI [−0.020, −0.003]), with two significant indirect effects in this model. The first examined whether Conflictive Climate in the Family mediated the relationship between perceived stress and life satisfaction, showing a negative relationship (b = −0.009, SE = 0.003, 95% CI [−0.016, −0.003]). The second indirect effect assessed whether Violent Climate mediated between perceived stress and life satisfaction, and it was also negative (b = −0.002, SE = 0.001, 95% CI [−0.006, −0.001]). However, the Supportive and Cohesive Climate and the Difficulty Expressing Feelings and Opinions climate factors did not significantly mediate the relationship between perceived stress and life satisfaction.

Finally, we analysed whether the significant indirect effects were differentially related in the group, comparing the strength of the indirect effects through pairwise contrasts. The effect of Conflictive Climate in the Family was slightly stronger than the mediating effect of Supportive and Cohesive Climate (b = −0.024, SE = 0.009, 95% CI [−0.043, −0.007]). Similarly, there was a significant difference with the factor Supportive and Cohesive Climate, slightly stronger than the mediating effect of Violent Climate (b = 0.010, SE = 0.005, 95% CI [0.002, 0.020]). In the rest of the pairwise comparisons of indirect effects, no significant differences were found.

## 4. Discussion

The present study examined the role of the family climate as a component of the dynamics of the family system in the relationship between perceived stress and life satisfaction, considered as personal characteristics. The research findings demonstrate that the mediating effect of family climate can contribute to understanding the relationship between perceived stress and life satisfaction in university students.

First, the results indicated that the perceived stress in our university participants was related to family climates. Particularly, there was a significant relationship between perceived stress and less conducive family climates, such as the Conflictive Climate and the Violent Climate, as posited in Hypothesis 1. For university students, the expectation of harmony within the family may heighten stress perception when tensions arise in parent–child relationships [52,53]. This familial relationship, in turn, can subject students to personal conflicts due to the pressure to please and not disappoint their parents. Bulo and Sánchez [33] asserted that university students believe stress impacts them more significantly when they experience academic failures, given that this is the primary reason for parental approval. Therefore, achieving high grades becomes a stress source that affects their success in creating a positive impression on their parents, classmates, and other significant individuals. Failures become pressures stemming from intrapersonal, interpersonal, academic, and environmental stress factors [33]. It was emphasized that relationships with friends and family can serve as both a moderate and extreme source of stress for young university students when they are not positive [34]. Rayle and Chung [54] affirmed that social and familial support aids in reducing stress among university students.

Empirical studies have suggested that the family climate can serve as a significant buffer against perceived stress by providing emotional support and resources to cope with external tensions [55]. Conger et al. [56] highlighted how stress levels can influence the quality of family interactions, which in turn can affect the family climate and cohesion. Other authors [57], have also found evidence that a positive family climate can mitigate the negative effects of perceived stress on individuals’ mental health. Family dynamics and perceived stress are inherently linked, with the family climate playing a crucial role in individuals’ ability to manage stress and maintain healthy family relationships.

Second, a significant relationship was also found between all family climates and life satisfaction, supporting Hypotheses 3 and 4. While the Conflictive and Violent Climates had indirect effects, the Cohesive Climate and the Difficulty Expressing Feelings climate had direct effects. Notably, the Conflictive Climate in the Family factor had the strongest relationship with life satisfaction.

The relationship with the family and satisfaction with this bond will influence the psychological and physical development of young individuals [13]. The presence of conflicts, communicative difficulties, lack of parental support, inappropriate educational models, etc., leads to a higher probability of disruptive behaviours, antisocial tendencies, and emotional problems such as anxiety or depression [31]. Conversely, parental support contributes to the life satisfaction of young individuals [58], and family conditions also play a role in this regard [59].

Regarding parenting style, it is noteworthy that warmth in educational approaches is positively related to high levels of satisfaction, contrasting with punishment-based approaches, which correlate negatively with life satisfaction [13]. Oberle et al. [58] argued that parental support plays an essential role in subjective well-being, like Leto et al. [59], who affirmed that family involvement in development affects life satisfaction, emphasizing the importance of parental warmth. Thus, if there is a positive family climate, the family can act as a protective factor: young individuals reporting good communication with their parents and a strong sense of family connection exhibited higher levels of life satisfaction and lower psychological distress [31]. A positive family atmosphere has been associated with life satisfaction in adolescents [60,61]; psychosocial adjustment; behaviour; social, physical, affective, and intellectual development [62]; and reduced depression and somatization [63]. Likewise, greater parental acceptance and lower psychological control predicted positive well-being and a lower likelihood of substance use during the transition to adulthood [64].

Vautero et al. [65] emphasized that the family environment influences not only academic outcomes and life satisfaction but also how young individuals perceive their future and, consequently, their life satisfaction. As Ni et al. [13] aptly stated, and as evidenced in this research, the family environment can significantly influence the personality development of young individuals and their assessment of subjective well-being.

Third, besides confirming the direct effects between stress and family climates, and between family climates and life satisfaction, this study explored the relationship between perceived stress and life satisfaction. There was a significant and indirect relationship between perceived stress and life satisfaction in our university sample, as stated in Hypothesis 5.

The inverse relationship between stress perception and life satisfaction has been consistently supported by research, as indicated by several empirical studies [21,66]. This phenomenon has been observed in diverse cohorts, encompassing not only university students but also military personnel and elderly migrants [67,68]. The interplay between stress perception and life satisfaction is often influenced by mediating factors such as self-efficacy [69], self-control, and rumination [21]. Zheng et al. [21] highlighted that self-control and rumination can play a partial mediating role in the connection between stress perception and life satisfaction, following a parallel pattern. Additionally, it was found that gratitude can moderate this relationship, especially between stress perception and life satisfaction in the military context [65]. In summary, facilitating the reduction of stress perception emerges as an effective strategy to enhance life satisfaction, supported by diverse empirical evidence across various populations.

Fourth, family climate can mediate the relationship between perceived stress and life satisfaction. The results indicated that this mediation is significant when considering Conflictive Climate in the Family and Violent Climate in the Family, allowing us to affirm Hypothesis 6, stating that the mediation of the family climate helps to better explain the relationship between perceived stress and life satisfaction.

Research on the relationship between perceived stress and life satisfaction has unveiled the potential mediation of family climate. Zarei and Fooladvand [70] identified that family functioning can have a significant direct impact on life satisfaction, and hope and resilience mediated this association. This underscores the importance of family functioning in individuals’ perception of well-being. Khurshid et al. [71] found that perceived stress acted as a mediator in the relationship between family social capital and life satisfaction, especially among working women. However, not all studies support a mediating effect of family climate. Szcześniak and Tułecka [72] discovered that while self-efficacy, self-control, and rumination partially mediated the connection between perceived stress and life satisfaction, family climate did not play a significant mediating role in this context. Overall, the complexity of the relationship between perceived stress and life satisfaction implies that various factors, including family climate, can influence this dynamic.

In conclusion, this study has demonstrated that the family climate acts as a significant mediator in the relationship between perceived stress and life satisfaction among university students. Our findings support the notion that a positive family climate characterized by cohesion and emotional support can mitigate the negative effects of perceived stress on life satisfaction [35,36].

By integrating Bronfenbrenner’s perspective [35], it is emphasized that the development and well-being of university students cannot be understood without considering the dynamic interactions between the individual and their broader familial, educational, and cultural environment. This ecological approach underscores the necessity for social policies that strengthen family and community support systems to foster healthy development and greater life satisfaction among youth.

Furthermore, the results highlight that different family climates, ranging from conflictive and violent to cohesive with difficulties in expressing emotions, play distinct roles in young adults’ life satisfaction [14]. These findings underscore the importance of considering these familial factors when designing interventions aimed at improving the psychological well-being of university students and promoting healthier family environments.

### Limitations

The present study has limitations. First, the cross-sectional design limits causal interpretation, although the results demonstrate some mediating relationships. Future studies could address this limitation by employing longitudinal designs, even within university/college contexts, to further explore these findings. Second, the generalizability of the results to all young students may be restricted due to variations in study types or cultural backgrounds. More heterogeneous samples are required. Third, social support, which helps to place family dynamics within a broader framework, was not measured in this study. Future research should assess peer support or social support, whether informal or formal, to gain a better understanding of the role of the family context in the personal characteristics of its members.

## 5. Conclusions

This study’s results yield valuable insights into the influence of family dynamics on the individual conditions of its members. It was identified that the family climate can play a mediating role in the relationship between perceived stress and life satisfaction among university students. Given the psychosocial changes inherent in youth and the unique characteristics of the university educational environment, individuals are more susceptible to stressful situations, which can significantly impact their life satisfaction. This influence extends beyond students’ academic development to encompass their familial and social spheres. Consequently, the findings underscore the importance of adopting a family-oriented approach to interventions targeting university students, particularly when confronted with elevated stress levels, as they have the potential to enhance overall life satisfaction.

## Figures and Tables

**Table 1:** Sociodemographic characteristics of the study sample.

Characteristics		n = 920
Age (Years) Mean (SD)		21.32 (3.54)
Sex [n, (%)]	Men	420 (45.7%)
	Women	500 (54.3%)
Branch of knowledge [n, (%)]	Arts and Humanities	98 (10.7%)
	Sciences and Health Sciences	153 (16.6%)
	Social and Legal Sciences	457 (49.7%)
	Engineering and Architecture	212 (23%)
Course [n, (%)]	First	240 (26.1%)
	Second	261 (28.4%)
	Third	251 (27.3%)
	Fourth	168 (18.3%)

**Table 2:** Descriptive statistics and correlation between measured variables.

Variables	M	SD	1	2	3	4	5	6
1. PS	26.44	7.44	-					
2. LS	18.45	3.41	−0.491 ***	-				
3. CCF	2.41	0.43	0.160 ***	−152 ***	-			
4. SCC	2.92	0.37	0.056	0.091 **	0.421 ***	-		
5. DEFO	3.07	0.52	−0.031	0.210 ***	0.187 ***	0.535 ***	-	
6. VCF	1.9	0.72	0.070 *	−153 ***	0.318 ***	0.071 *	−0.100 **	-

M = mean; SD = standard deviation; PS = perceived stress; LS = life satisfaction. Family climates: CCF, Conflictive Climate in the Family; SCC, Supportive and Cohesive Climate; DEFO, Difficulty Expressing Feelings and Opinions; VCF, Violent Climate in the Family. * *p* < 0.05, ** *p* < 0.01, *** *p* < 0.001.

**Table 3:** Direct, indirect, and total effects of perceived stress on life satisfaction mediated by family climates (parallel mediation).

Model Path	Effect	Boot-LLCI	Boot-ULCI	SE/BootSE *	t	*p*-Value
Direct effects						
PS → LS	−0.214	−0.239	−0.188	0.013	−16.598	0.000
Indirect effects (**)						
PS → CCF → LS	−0.009	−0.016	−0.003	0.003		Y
PS → SCC → LS	0.002	−0.000	0.006	0.002		N
PS → DEFO → LS	−0.002	−0.007	0.002	0.002		N
PS → VCF → LS	−0.002	−0.006	−0.001	0.001		Y
Total indirect effects	−0.011	−0.020	−0.003	0.004		Y
Total effects	−0.225	−0.251	−0.199	0.013	−17.074	0.000

Note: PS, perceived stress; LS, life satisfaction. Family climates: CCF, Conflictive Climate in the Family; SCC, Supportive and Cohesive Climate; DEFO, Difficulty Expressing Feelings and Opinions; VCF, Violent Climate in the Family. (*) The indirect effect does not include the T or *p*-value. (**) The indirect effect will be significant if the bootstrapping interval does not contain 0 (Y, significant; N, not significant).

## Data Availability

All data generated or analysed during this study are included in this published article.
